# Comparison of the Clinical Value of miRNAs and Conventional Biomarkers in AMI: A Systematic Review

**DOI:** 10.3389/fgene.2021.668324

**Published:** 2021-06-17

**Authors:** Baofu Wang, Yang Li, Xuezeng Hao, Jingjing Yang, Xiaowan Han, Haiyan Li, Tong Li, Dayang Wang, Yu Teng, Liang Ma, Yao Li, Mingjing Zhao, Xian Wang

**Affiliations:** ^1^Dongzhimen Hospital of Beijing University of Chinese Medicine, Beijing, China; ^2^Institute of Cardiovascular Diseases, Beijing University of Chinese Medicine, Beijing, China

**Keywords:** miRNAs, conventional biomarkers, acute myocardial infarction, peak time, AUC

## Abstract

**Background/Aims:** This study aimed to compare the clinical value of the peak time point and area under the curve (AUC) of miRNAs and conventional biomarkers in acute myocardial infarction (AMI).

**Methods:** A literature search was carried out in PubMed, Web of Science, Embase, and Cochrane systematically. Screening studies, extracting data, and assessing article quality were performed independently by two researchers. Also, the names of miRNAs in the included studies were standardized by the miRBase database.

**Results:** A total of 40 studies, encompassing 6,960 participants, were included in this systematic review. The samples of circulating miRNAs were mainly from the plasma. The results of this systematic review displayed that miR-1-3p, miR-19b-3p, miR-22-5p, miR-122-5p, miR-124-3p, miR-133a/b, miR-134-5p, miR-150-5p, miR-186-5p, miR-208a, miR-223-3p, miR-483-5p, and miR-499a-5p reached peak time earlier and showed a shorter time window than the conventional biomarkers despite the different collection times of initial blood samples. miR-1-3p, miR-19b-3p, miR-133a/b, miR-208a/b, miR-223-3p, miR-483-5p, and miR-499a-5p were shown to be more valuable than classical biomarkers for the early diagnosis of AMI, and these miRNAs appeared to have the most potential biomarkers within 4 h of the onset of symptoms except miR-133a/b and miR-208b. Moreover, combined miRNAs or miRNAs combined with classical biomarkers could compensate for the deficiency of single miRNA and conventional biomarker in sensitivity or specificity for an optimal clinical value.

**Conclusions:** miR-1-3p, miR-19b-3p, miR-208a, miR-223-3p, miR-483-5p, and miR-499a-5p are promising biomarkers for AMI due to their satisfactory diagnostic accuracy and short time window (within 4 h of the onset of symptoms).

## Introduction

Acute myocardial infarction (AMI) accounted for the major proportion of morbidity and mortality in coronary heart disease (CHD) (White and Chew, 2008). Early diagnosis can prevent and alleviate cardiac cell death, improve heart function, and reduce cardiovascular adverse events. Conventional blood biomarkers, such as cardiac troponin (cTn), creatine kinase MB (CKMB), and high-sensitivity cTnT (hs-cTnT), are regarded as gold standards and widely used to diagnose AMI (Thygesen et al., 2018). However, circulating cTn is released slowly such that the concentration cannot be detected immediately in the early phase of AMI (Baker et al., 2011). In addition, these biomarkers have some limitations in specificity. Some non-AMI diseases, including myopericarditis, acute heart failure, stable chronic heart failure, acute pulmonary embolism, and chronic kidney disease, can falsely elevate cTn (Giannitsis and Katus, 2013; Thygesen et al., 2018). Thus, a novel diagnostic biomarker is necessary to meet the clinical demands.

MicroRNAs (miRNAs) are endogenous small non-coding RNAs that play a major role in various physiological and pathological processes (Lalem and Devaux, 2019). Due to their stability and tissue/cell specificity in peripheral circulation (Mitchell et al., 2008; D'Alessandra et al., 2010), a large number of circulating miRNAs have been reported as new potential biomarkers for diagnosing AMI (Zhao et al., 2019; Su J. et al., 2020; Wexler and Nussinovitch, 2020). However, whether miRNAs have a similar or equal clinical value with traditional biomarkers is yet to be elucidated. Therefore, this systematic review was conducted to compare the time window and area under the curve (AUC) of miRNAs and conventional biomarkers (cTnI/cTnT/CKMB/hs-cTnT).

## Methods

### Search Strategy

Articles published before September 9, 2020, were searched comprehensively in electronic databases (PubMed, Web of Science, Embase and Cochrane) using the search terms “myocardial infarction” and “microRNAs” and their common synonyms.

### Inclusion and Exclusion Criteria

The inclusion criteria of the studies were as follows: ① participants in the case group met the diagnostic criteria of AMI/ST-segment elevation myocardial infarction (STEMI)/non-ST-segment elevation myocardial infarction (NSTEMI) and the control group was non-AMI, including healthy volunteers or subjects without MI/AMI/cardiovascular disease; ② items were related to peak hour comparison of miRNAs and conventional biomarkers or AUC comparison of miRNAs and conventional biomarkers or peak hour and AUC comparison of miRNAs and conventional biomarkers; ③ the samples were from the plasma or serum. The exclusion criteria were as follows: ① non-clinical study; ② articles with incomplete information; ③ reviews, meta-analyses, and corresponding/conference abstracts.

### Data Extraction

Titles and abstracts of all included studies were assessed independently by two researchers (BW and YL) according to the inclusion and exclusion criteria. Data, including first author's name, year of publication, country, inclusion criteria, sample size, age, gender, medical history, miRNAs, classical biomarkers, detection method, detection time points, peak point, AUC, sensitivity, and specificity, were extracted from the included studies. The names of miRNAs were also standardized through the miRBase database. Any disagreement was resolved by discussing and consulting with the corresponding authors (MZ and XW).

### Quality Assessment

The Newcastle–Ottawa scale (NOS) was used to assess the quality of studies based on three factors: the selection of the research population, compatibility of the study groups, and measurement of exposure factors. Each study scored 0–9 points.

### Summary Analysis

A qualitative synthesis was adopted for this systematic review.

## Results

### Literature Search Results

**Figure 1:**
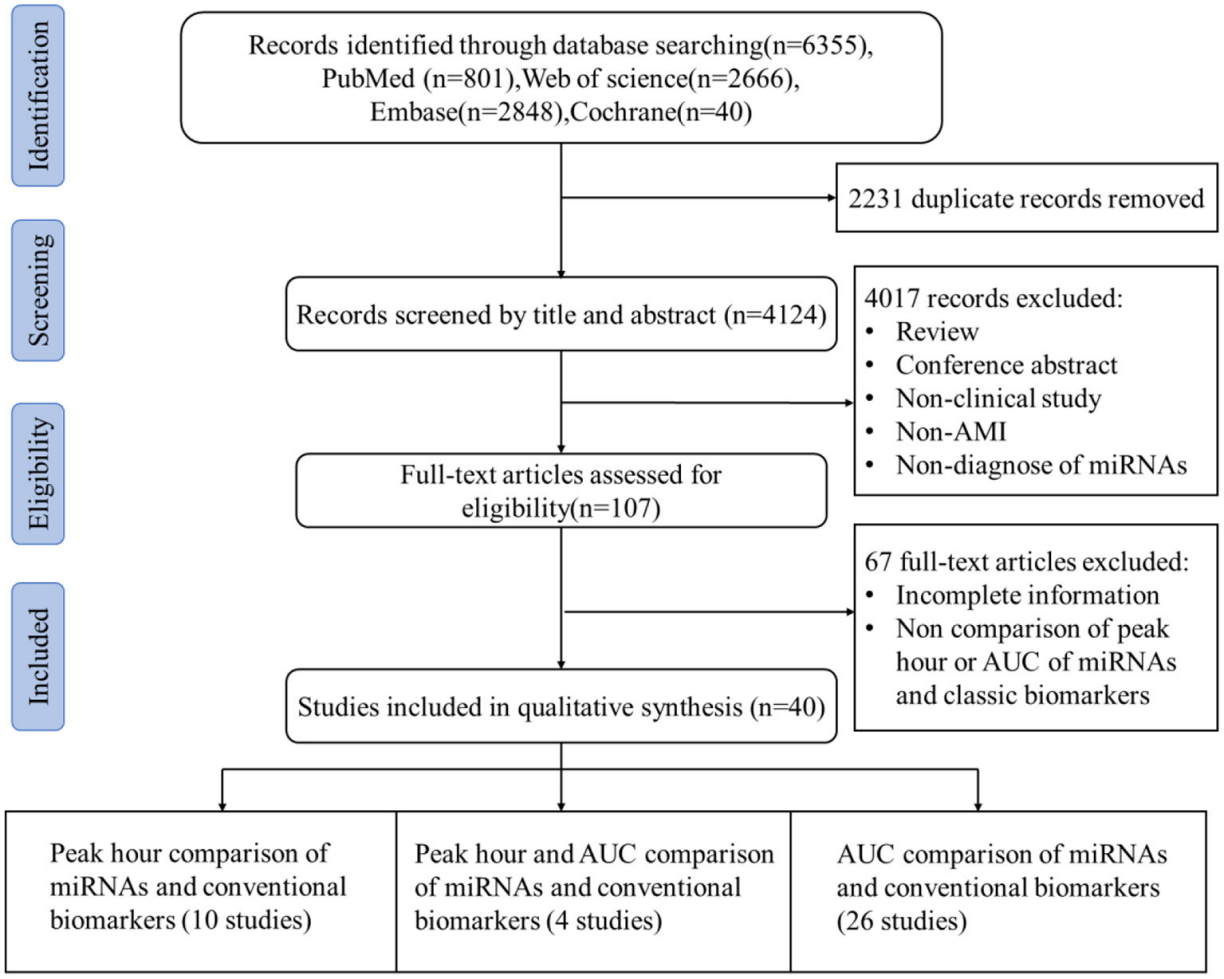
Flow diagram of the literature search.

A total of 6,355 records were identified from four electronic databases, and 2,231 duplicate articles were removed. Subsequently, 4,017 studies were excluded after screening titles and abstracts, and 107 articles were subjected to full-text analysis. Finally, 40 studies that fulfilled the selection criteria were divided into three categories as follows, and the specific filtering process was illustrated in Figure 1.

① Peak hour comparison of miRNAs and conventional biomarkers (10 studies).
② AUC comparison of miRNAs and conventional biomarkers (26 studies).
③ Peak hour and AUC comparison of miRNAs and conventional biomarkers (4 studies).

### Study and Patient Characteristics

Overall, 6,960 participants were included based on the criteria of non-AMI (healthy volunteers or those without MI/AMI/cardiovascular disease) and AMI/STEMI/NSTEMI. A majority of studies focused on the miRNAs in the plasma, followed by the serum (Supplementary Tables 2–4), and qPCR was used to detect miRNAs. Also, the names of miRNAs were standardized by the miRBase database (Supplementary Table 1). The data of study ID, inclusive criteria, sample size, peak hour, and AUC of miRNAs and classical biomarkers were extracted for further evaluation.

1. Peak hour comparison of miRNAs and conventional biomarkers: As shown in Table 1, 561 participants from China, Portugal, Italy, and Poland were included. Notably, the time of collecting initial blood samples varied. The first samples were gained from the onset of symptoms/admission/cardiac catheterization, and the detection time interval was also different.
2. AUC comparison of miRNAs and conventional biomarkers: Participants who met the inclusive criteria were recruited in the study. The majority of the studies focused on the comparison between miR-1-3p/miR-133a/miR-208a/miR-208b/miR-499-5p and cTnT/cTnI, but only a few studies addressed the comparison of sensitivity and specificity (Table 2).
3. Peak hour and AUC comparison of miRNAs and conventional biomarkers: 349 participants diagnosed with AMI and 292 participants with non-AMI were enrolled in the studies (Table 3A). Three studies used plasma to detect miRNAs and classical biomarkers, while one study used plasma to detect miRNAs and serum for classical biomarkers. Although the first samples were obtained from the onset of symptoms, the detection time interval was also different. Furthermore, the comparison of AUC only referred to miRNAs and cTnI/CKMB (Table 3B).

**Table 1:** Peak hour comparison of miRNAs and conventional biomarkers.

**Study ID**	**Inclusive criteria**		**Detection time point**	**miRNA**	**Peak hour**				
**Author/year/country**	**Control group (N)**	**Case group (N)**			**miRNA**	**cTnI**	**hs-cTnT**	**cTnT**	**CKMB**
D'Alessandra et al. (2010)/ Italy	non-AMI (17)	STEMI (8)	T0^1^, 3, 9, 15, 21, 33, 45, and 69 h	miR-1-3p	T0^1^	3 h			
				miR-133a	T0^1^				
				miR-133b	T0^1^				
				miR-499-5p	9 h				
Białek et al. (2015)/ Poland	non-AMI (8)	STEMI (19)	T0^2^, 3, 6, 12, and 24 h	miR-208a	3 h	6 h			6 h
Guo et al. (2017)/ China	non-AMI (45)	AMI (90)	T0^2^, 6, 12, and 24 h	miR-124-3p	6 h	12 h			12 h
Li H. et al. (2019)/ China	non-AMI (55)	AMI (35)	T0^3^, 4 h (±30 min), 12 h (±30 min), 24 h (±30 min), 48 h (±30 min), and 72 h (±30 min)	miR-22-5p	T0^3^	12 h			
				miR-132-5p	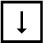				
				miR-150-5p	T0^3^				
Wang et al. (2016)/ China	non-AMI (20)	AMI (18)	T0^4^, 4 h (±30 min), 8 h (±30 min), 12 h (±30 min), 24 h (±30 min), 48 h (±30 min), and 72 h (±30 min)	miR-19b-3p	T0^4^	8 h			
				miR-134-5p	T0^4^				
				miR-186-5p	4 h				
Cortez-Dias et al. (2016)/ Portugal	non-AMI (18)	STEMI (40)	T0^5^, 8, 16, 24, 48, and 72 h after cardiac catheterization	miR-1-3p	8 h	8 h			8 h
				miR-122-5p	T0^5^				
				miR-133a-3p	8 h				
				miR-133b	8 h				
				miR-208b-3p	8 h				
				miR-499a-5p	8 h				
Long et al. (2012a)/ China	non-AMI (25)	AMI (17)	T0^6^, 4 h (±30 min), 8 h (±30 min), 12 h (±30 min), 24 h (±30 min), 48 h (±30 min), 72 h (±30 min), and 1 week (±60 min)	miR-1-3p	8 h	8 h			
				miR-126	8 h				
Long et al. (2012b)/ China	non-AMI (30)	AMI (18)	T0^6^, 4 h (±30 min), 8 h (±30 min), 12 h (±30 min), 24 h (±30 min), 48 h (±30 min), 72 h (±30 min), and 1 week (±60 min)	miR-30a-5p	8 h	8 h			
				miR-195-5p	8 h				
				let-7b-5p	8 h				
Li Z. et al. (2014)/ China	non-AMI (31)	AMI (27)	T0^6^, 4 h (±30 min), 8 h (±30 min), 12 h (±30 min), 24 h (±60 min), 48 h (±60 min), and 72 h (±60 min)	miR-497-5p	8 h	8 h			
Wang et al. (2013)/ China	non-AMI (27)	AMI (13)	T0^7^, 4, 12, 24, 48, 72 h	miR-133a	4 h	4 h			

*non-AMI, healthy volunteers or subjects without MI/AMI/cardiovascular disease; AMI, patients with AMI/STEMI/NSTEMI; N, sample size. The samples were obtained from or after T0. T0^1^, the samples were obtained at 2.6 ± 1.2 h after the onset of MI symptoms; T0^2^, admission time; T0^3^, the samples were obtained at 9.24 ± 2.81 h after the onset of AMI symptoms; T0^4^, the samples were obtained at 10.40 ± 3.52 h after the onset of chest pain symptoms; T0^5^, the samples were obtained after cardiac catheterization; T0^6^, the time of the onset of symptoms; T0^7^, the samples were obtained at 17.6 ± 4.5 h after onset of AMI; 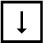, sustainable low level at all time points*.

**Table 2:** AUC comparison of miRNAs and conventional biomarkers.

**Study ID**	**Inclusive criteria**		**miRNA**	**AUC/Sensitivity/Specificity**					
**Author/year/ country**	**Control group (N)**	**Case group (N)**		**miRNA**	**Combination value**	**cTnI**	**hs-cTnT**	**cTnT**	**CKMB**
Agiannitopoulos et al. (2018)/ Greek	non-AMI (50)	AMI (80)	miR-208b	0.999/98%/100%				0.940	
			miR-499a-5p	0.999/98%/100%					
Liu et al. (2018)/ China	non-AMI (30)	NSTEMI (145)	miR-1-3p	0.773			0.778		
			miR-133	0.928					
			miR-208a-3p	0.994					
			miR-499a-5p	0.994					
Vengatapathy et al. (2019)/ India	non-AMI (60)	AMI (60)	miR-499a-5p	0.974/93.33%/86.67%			0.924/90%/81.56%		0.893/86.67%/71.67%
Devaux et al. (2012)/ Luxembourg	non-AMI (87)	STEMI; NSTEMI (510)	miR-208b	0.9/79%/100%			0.97/93%/98%		
			miR-499a-5p	0.97/95%/100%					
Zhong et al. (2014)/ China	non-AMI (145)	AMI (156)	miR-19a-3p	0.997			0.717		0.511
Zhang et al. (2018)/ China	non-AMI (60)	STEMI (80)	miR-23b-3p	0.809		0.783			0.753
He et al. (2017)/ China	non-AMI (30)	AMI (27)	miR-126-3p	0.992/96.7%/93.3%		0.787			0.863
Zhu et al. (2016)/ China	non-AMI (60)	AMI (60)	miR-181a-5p	0.834/89.8%/82.6%		0.873		0.816	
Su T. et al. (2020)/ China	non-AMI (163)	AMI (174)	miR-1-3p	0.863/87.9%/80.4%		0.862/79.3%/91.4%			0.719/57.5%/94.5%
Zhang et al. (2016)/ China	non-AMI (10)	AMI (17)	miR-21-5p	0.892		1.000			0.66
Su et al. (2019)/ China	non-AMI (167)	AMI (174)	miR-1-3p	0.863/87.9%/80.2%	0.931/86.2%/90.4%^*^	0.864/79.3%/91.6%			
Wang et al. (2010)/ China	non-AMI (33)	AMI (33)	miR-1-3p	0.847/33.3%/100%		0.987			
			miR-133a	0.867/15.2%/100%					
			miR-208a	0.965/90.9%/100%					
			miR-499a-5p	0.822/36.4%/100%					
Li C. et al. (2013)/ China	non-AMI (100)	AMI (117)	miR-1-3p	0.696	0.811^#^			0.800	0.683
			miR-134	0.657					
			miR-186-5p	0.715					
			miR-208a-3p	0.778					
			miR-223-3p	0.741					
			miR-499a-5p	0.755					
Li Y. Q. et al. (2013)/ China	non-AMI (32)	AMI (67)	miR-1-3p	0.826				0.982	
			miR-133a	0.9468					
			miR-208b	0.8899					
			miR-499a-5p	0.884					
Li L. M. et al. (2014)/ China	non-AMI (28)	AMI (56)	miR-1-3p	0.854				0.959	
Robinson et al. (2018)/ Germany	non-AMI (20)	STEMI (24)	miR-21-5p	0.6083		0.9432			
			miR-208a	0.6917					
			miR-499a-5p	0.8417					
Dai et al. (2020)/ China	non-AMI (50)	AMI (88)	miR-32-5p	0.949/92%/84%		0.993			
Zhang et al. (2017)/ China	non-AMI (35)	AMI (42)	miR-92a-3p	0.888		0.912			
Ke-Gang et al. (2016)/ China	non-AMI (79)	AMI (233)	miR-133a	0.667/61%/68%		0.964/90%/90%			0.867/74%/93%
Yuan et al. (2016)/ China	non-AMI (110)	AMI (102)	miR-133a	0.870		0.881			0.778
He et al. (2014)/ China	non-AMI (30)	AMI (359)	miR-134	0.818/79%/77.1%			0.962/82.1%/92.5%		
			miR-328	0.887/86.3%/74.6%					
Coskunpinar et al. (2016)/ Turkey	non-AMI (16)	AMI (27)	miR-221-3p	0.881		0.954			
Li P. et al. (2019)/ China	non-AMI (10)	AMI (41)	miR-208a-3p	0.868			0.992		
			miR-494	0.839					
			miR-499a-5p	0.852					
			miR-1303	0.884					
Gidlöf et al. (2013)/ Sweden	non-AMI (88)	STEMI; NSTEMI (319)	miR-1-3p	0.57				0.95	
			miR-208b	0.82					
			miR-499a-5p	0.79					
Devaux et al. (2015)/ Luxembourg	non-AMI (931)	AMI (224)	miR-208b	0.76/64.7%/80.2%	0.94^*^		0.94		
					0.86^*^			0.84	
			miR-499a-5p	0.65/35.7%/90.3%	0.94^*^		0.94		
					0.84^*^			0.84	
Zhao et al. (2015)/ China	non-AMI (60)	AMI (59)	miR-499a-5p	0.915/86.37%/93.47%		0.971/93.12%/100%			

*non-AMI: healthy volunteers, or subjects without MI/AMI/cardiovascular disease; AMI: patients with AMI/STEMI/NSTEMI; N: sample size;*

* *the AUC of combination of miRNAs with classical biomarkers*.

# *the AUC of combined miRNAs*.

**Table 3A:** Peak hour and AUC comparison of miRNAs and conventional biomarkers.

**Study ID**	**Inclusive criteria**		**Detection time point**	**miRNA**	**Peak hour**				
**Author/year/ country**	**Control group (N)**	**Case group (N)**			**miRNA**	**cTnI**	**hs-cTnT**	**cTnT**	**CKMB**
Li L. et al. (2019)/ China	non-AMI (140)	AMI (140)	T0^8^, 0–3, 3–7, 7–11, 11–15, 15–19, 19–23, 23–27, and 27–31 h after onset of chest pain	miR-19b-3p	T0^8^	16–24 h			16–24 h
				miR-223-3p	T0^8^				
				miR-483-5p	T0^8^				
Zhang et al. (2015)/ China	non-AMI (85)	AMI (142)	T0^9^, 0–3, 3–6, 6–9, and >9 h after the onset of chest pain	miR-499a-5p	6–9 h	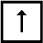			6–9 h
Wang et al. (2014)/ China	non-AMI (28)	AMI (17)	T0^10^, 4, 12, 24, 48, and 72 h	miR-21-5p	4 h	4 h			
				miR-361-5p	4 h				
				miR-519e-5p	24 h 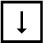				
Yao et al. (2015)/ China	non-AMI (39)	AMI (50)	T0^11^, 4, 8, 12, and 24 h	miR-122-5p	8 h	8 h			

**Table 3B:** Peak hour and AUC comparison of miRNAs and conventional biomarkers.

**AUC/Sensitivity/Specificity**					
**miRNAs**	**Combination miRNAs**	**cTnI**	**hs-cTnT**	**cTnT**	**CK-MB**
0.74^*^; 0.81/76%/88%^**^	0.81^*#^; 0.89/78%/92%^*##^	0.77^*^; 0.68/48%/98%^**^			0.73^*^
0.65^*^; 0.76/51%/94%^**^					
0.7^*^; 0.78/58%/96%^**^					
0.86/80%/80.28%		0.90			0.82
0.949, 0.947, 0.791^***^	0.989, 1.000, 0.995^#^	1.000, 1.000, 1.000^***^			
0.881, 0.883, 0.838^***^					
0.798, 0.801, 0.908^***^					
0.855		0.902			

*The samples were obtained from or after T0. T0^8^, the samples were obtained at 2.2 ± 0.97 h after the onset of chest pain on admission; T0^9^, the time of chest pain; T0^10^, the samples were obtained at 12.4 ± 1.5 h after the onset of AMI symptoms; T0^11^, the time of onset of AMI; 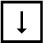, lowest time; 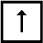, sustain increase;*

* *the AUC of miRNAs or classical biomarkers in patients with AMI (including all time points);*

** *the AUC of miRNAs or classical biomarkers in patients with chest pain for <3 h;*

*# *the AUC of combined miRNAs in patients with AMI (including all time points);*

*## *the AUC of combined miRNAs in patients with chest pain for <3 h;*

*** *the AUC of miRNAs or classical biomarkers at T0 and 4 and 24 h;*

# *the AUC of combination of miRNAs at T0 and 4 and 24 h*.

### Quality Assessment of Included Studies

The mean score of NOS in the included studies was 7.05. The quality assessment of the included studies was described in the Supplementary Table 5.

### Peak Hour Comparison of miRNAs and Conventional Biomarkers

As shown in Table 1, most miRNAs showed a satisfactory time window for identifying the early phase of AMI despite different sampling and detecting time points. The level of miR-1-3p, miR-19b-3p, miR-22-5p, miR-122-5p, miR-124-3p, miR-133a, miR-133b, miR-134-5p, miR-150-5p, miR-186-5p, and miR-208a was dynamically detected from T0. miR-1-3p, miR-19b-3p, miR-22-5p, miR-122-5p, miR-133a, miR-133b, miR-134-5p, and miR-150-5p achieved peak immediately at T0 while miR-124-3p, miR-186-5p, and miR-208a expressions reached the peak levels at 6, 4, and 3 h, respectively. These miRNAs reached the peak expression 3–12 h earlier than cTnI/CKMB in the early phase of AMI (D'Alessandra et al., 2010; Białek et al., 2015; Cortez-Dias et al., 2016; Wang et al., 2016; Guo et al., 2017; Li H. et al., 2019).

Other publications revealed that miRNAs could be detected concurrently to, or later than, conventional biomarkers. Five studies showed that miRNAs, including let-7b-5p, miR-1-3p, miR-30a-5p, miR-126, miR-133a, miR-133a-3p, miR-133b, miR-195-5p, miR-208b-3p, miR-497-5p, and miR-499a-5p exhibited a similar trend to that of traditional biomarkers and achieved a peak at the same time points (Long et al., 2012a,b; Wang et al., 2013; Li L. M. et al., 2014; Cortez-Dias et al., 2016). Wang et al. (2013) collected blood samples at 4, 12, 24, 48, and 72 h after T0, and the results showed that circulating miR-133a and cTnI increased and achieved a peak at 4 h. Four other studies revealed that let-7b-5p, miR-1-3p, miR-30a-5p, miR-126, miR-133a-3p, miR-133b, miR-195-5p, miR-208b-3p, miR-497-5p, and miR-499a-5p were highly expressed in AMI compared to the control group and reached the peak expression at 8 h, which was similar to that of cTnI (Long et al., 2012a,b; Li Z. et al., 2014; Cortez-Dias et al., 2016) and CKMB (Cortez-Dias et al., 2016). In addition, miR-132-5p displayed a sustainable low level at all time points, and miR-499a-5p showed a peak level (9 h) later than that of cTnI (3 h) (D'Alessandra et al., 2010; Li H. et al., 2019).

### AUC Comparison of miRNAs and Conventional Biomarkers

#### AUC Comparison of Single miRNA and Traditional Biomarker

As shown in Table 2, some studies reported that miRNAs had a better accuracy than the classical biomarkers. Agiannitopoulos et al. (2018) found that the AUC of both miR-208b and miR-499a-5p was 0.999, which was slightly higher than that of cTnT (0.94). Liu et al., Vengatapathy et al., and Devaux et al. showed that the diagnostic value of miR-208a-3p [0.994 (Liu et al., 2018)] and miR-499a-5p [0.994 (Liu et al., 2018), 0.974 (Vengatapathy et al., 2019), and 0.97 (Devaux et al., 2012)] was no less than that of hs-cTnT [0.778 (Liu et al., 2018), 0.924 (Vengatapathy et al., 2019), and 0.97 (Devaux et al., 2012)]. miR-133 and miR-19a-3p also showed a satisfactory diagnostic value, and the AUC of miR-133 and miR-19a-3p was 0.928 (Liu et al., 2018) and 0.997 (Zhong et al., 2014), respectively, compared to that of hs-cTnT [0.778 (Liu et al., 2018) and 0.717 (Zhong et al., 2014), respectively]. In addition, miR-23b-3p, miR-126-3p, and miR-181a-5p also showed a more accurate AUC. The AUC for miR-23b-3p, miR-126-3p, and miR-181a-5p was 0.809 (Zhang et al., 2018), 0.992 (He et al., 2017), and 0.834 (Zhu et al., 2016), respectively, which were higher than those of cTnI [0.783 (Zhang et al., 2018) and 0.787 (He et al., 2017)] and cTnT [0.816 (Zhu et al., 2016)]. Moreover, most of the studies revealed that the AUC of miRNAs was also superior to that of CKMB. As shown in Table 2, the AUC of miRNAs was 0.013–0.486 higher than that of CKMB, especially miR-1-3p, miR-19a-3p, miR-21-5p, and miR-126-3p, which were 0.144 (Su T. et al., 2020), 0.486 (Zhong et al., 2014), 0.232 (Zhang et al., 2016), and 0.129 (He et al., 2017) higher than that of CKMB.

There were also studies that revealed different results. The AUC of miRNAs was lower than that of cTnI, cTnT, and hs-cTnT, but yet satisfactory. In Table 2, the AUC of miR-1-3p was 0.773 (Liu et al., 2018), 0.863 (Su et al., 2019; Su T. et al., 2020), 0.847 (Wang et al., 2010), 0.696 (Li C. et al., 2013), 0.826 (Li Y. Q. et al., 2013), and 0.854 (Li L. M. et al., 2014), which resembled that of cTnI [0.862 (Su T. et al., 2020) and 0.864 (Su et al., 2019)] and hs-cTnT [0.778 (Liu et al., 2018)] or was lower than that of cTnI [0.987 (Wang et al., 2010)] and cTnT [0.800 (Li C. et al., 2013), 0.982 (Li Y. Q. et al., 2013), and 0.959 (Li L. M. et al., 2014)]. The results of the AUC comparison of miR-21-5p (Zhang et al., 2016; Robinson et al., 2018), miR-32-5p (Dai et al., 2020), miR-92a-3p (Zhang et al., 2017), miR-133a (Wang et al., 2010; Li Y. Q. et al., 2013; Ke-Gang et al., 2016; Yuan et al., 2016), miR-134 (Li C. et al., 2013; He et al., 2014), miR-181a-5p (Zhu et al., 2016), miR-186-5p (Li C. et al., 2013), miR-221-3p (Coskunpinar et al., 2016), miR-223-3p (Li C. et al., 2013), miR-208a-3p (Li C. et al., 2013; Li P. et al., 2019), miR-208a (Wang et al., 2010; Robinson et al., 2018), miR-208b (Devaux et al., 2012, 2015; Gidlöf et al., 2013; Li Y. Q. et al., 2013), miR-494 (Li P. et al., 2019), miR-499a-5p (Wang et al., 2010; Gidlöf et al., 2013; Li C. et al., 2013; Li Y. Q. et al., 2013; Devaux et al., 2015; Zhao et al., 2015; Robinson et al., 2018; Li P. et al., 2019), miR-328 (He et al., 2014), and miR-1303 (Li P. et al., 2019) with traditional biomarkers were similar to those with miR-1-3p.

#### AUC of Combined miRNA Was Compared to That of Individual miRNA or Classical Biomarker

Table 2 showed that the combination miRNAs or combination miRNAs with classical biomarkers increased the AUC of single miRNAs and traditional biomarkers. According to the publications, Li C. et al. (2013) demonstrated that the combination of miR-1-3p, miR-134, miR-186-5p, miR-208a-3p, miR-223-3p, and miR-499a-5p increased the AUC to 0.811, which was higher than that of the single miRNAs (miR-1-3p: 0.696; miR-134: 0.657; miR-186-5p: 0.715; miR-208a-3p: 0.778; miR-223-3p: 0.741; and miR-499a-5p: 0.755) and cTnT (0.800). Besides that, the diagnostic value of combined miRNAs and classical biomarkers was also increased. The AUC of the combination of miR-1-3p with cTnT, miR-208b with hs-cTnT, miR-208b with cTnT, miR-499a-5p with hs-cTnT, and miR-499a-5p with cTnT was 0.931, 0.94, 0.86, 0.94, and 0.84, respectively, which were significantly higher than that of single miRNA [miR-1-3p: 0.863 (Su et al., 2019); miR-208b: 0.76 (Devaux et al., 2015); and miR-499a-5p: 0.65 (Devaux et al., 2015)], cTnI [0.864 (Su et al., 2019)], and cTnT [0.84 (Devaux et al., 2015)], while the AUC of the combination of miR-208b with hs-cTnT (0.94) and miR-499a-5p with hs-cTnT (0.94) was identical to that of hs-cTnT (0.94) (Devaux et al., 2015).

#### Comparison of the Sensitivity and Specificity for miRNAs and Conventional Biomarkers

A few studies compared the sensitivity and specificity of miRNAs and conventional biomarkers. In the study by Su et al., miR-1-3p had a better sensitivity but lower specificity [87.9 and 80.4% (Su T. et al., 2020); 87.9 and 80.2% (Su et al., 2019)] than cTnI [79.3 and 91.4% (Su T. et al., 2020); 79.3 and 91.6% (Su et al., 2019)] and CKMB [57.5 and 94.5% (Su T. et al., 2020)]. In the combination of miR-1-3p and cTnT, the specificity could be increased to 90.4% with a stable sensitivity (86.2%), higher than the specificity of miR-1-3p (80.2%) and the sensitivity of cTnI (79.3%) (Su et al., 2019). Furthermore, the study of Devaux et al. (2012) demonstrated that the specificity of miR-208b or miR-499a-5p could reach 100% while that of hs-cTnT was 98% and that the sensitivity of miR-499a-5p (95%) was also slightly higher than that of hs-cTnT (93%). Other studies also reported that the specificity of miR-1-3p, miR-133a, miR-208a, miR-208b, or miR-499a-5p could reach 100% with a satisfactory sensitivity, but there were no references of traditional biomarkers (Wang et al., 2010; Agiannitopoulos et al., 2018). Overall, 13 studies were involved, and the range of specificity of most miRNAs was 80–100%, and that of sensitivity was 79–100% (Table 2).

### Peak Hour and AUC of miRNAs Were Compared With Conventional Biomarkers Simultaneously

A total of four studies reported the peak hour and AUC of miRNAs and classical biomarkers simultaneously. As shown in Table 3, Li L. et al. (2019) reported that the levels of miR-19b-3p, miR-223-3p, and miR-483-5p were significantly increased in patients with AMI, and the highest concentration was at T0 while cTnI and CKMB reached the peak level at 16–24 h after T0. The AUC of miR-19b-3p, miR-223-3p, and miR-483-5p was 0.74, 0.65, and 0.7, respectively, lower than that of cTnI (0.77). However, the AUC of the combination of these three miRNAs could get an incremental value (0.81) for diagnosis of AMI (including subjects with chest pain for <3, 3–6, and ≥6 h), which was higher than that of cTnI (0.77) and CKMB (0.73). Furthermore, the present study demonstrated that these three miRNAs have an optimal AMI diagnostic value in patients with chest pain for <3 h. The data showed that the AUC for miR-19b-3p, miR-223-3p, miR-483-5p, and the miRNA panel (a combination of these three miRNAs) was 0.81, 0.76, 0.78, and 0.89, respectively, and each miRNA had better diagnostic accuracy compared with cTnI (0.68). Meanwhile, the sensitivity of miR-19b-3p, miR-223-3p, miR-483-5p, and the miRNA panel was 76, 51, 58, and 78%, respectively, higher than that of cTnI (48%). Also, the specificity of miR-19b-3p (88%), miR-223-3p (94%), miR-483-5p (96%), and miRNA panel (92%) was satisfactory. Notably, 76.1 and 77.5% of all AMI patients with chest pain for <3 h were detected positive by miR-19b-3P and the miRNA panel, respectively, which was significantly higher than the figure of cTnI (47.8%).

Other studies reported that the expression of circulating miR-21-5p, miR-122-5p, miR-361-5p, and miR-499a-5p was significantly increased while miR-519e-5p had a remarkably reduced expression and exhibited the lowest concentration at 24 h after T0 (Wang et al., 2014; Yao et al., 2015; Zhang et al., 2015). miR-21-5p, miR-122-5p, miR-361-5p, and miR-499a-5p reached their peak expression at 4, 8, 4, and 6–9 h, respectively, and the peak time of miR-21-5p, miR-122-5p, and miR-361-5p was similar to that of cTnI (Wang et al., 2014; Yao et al., 2015; Zhang et al., 2015). However, the AUC of these miRNAs (miR-21-5p, miR-122-5p, miR-361-5p, miR-519e-5p, and miR-499a-5p) was not superior to that of cTnI. As shown in Table 3B, the diagnostic value of these miRNAs was similar to that of cTnI and CKMB, whereas the AUC of miR-21-5p and miR-361-5p decreased with prolonged detection time. The optimal diagnostic value of the combination of miRNA-21-5p, miRNA-361-5p, and miRNA-519e-5p was at T4 (1.00), while cTnI showed a stable diagnostic value at T0 (1.00), T4 (1.00), and T24 (1.00) (Wang et al., 2014).

## Discussion

AMI is one of the leading causes of death worldwide, and an early diagnostic marker is crucial and imperative for the efficient and timely therapy of AMI. cTn and CKMB are currently regarded as the critical biomarkers in diagnosing AMI. However, their clinical value for diagnosing early AMI and distinguishing it from non-AMI diseases remains limited (Feng et al., 2008; Moe and Wong, 2010; Thygesen et al., 2018). cTn and CKMB are released from cardiomyocytes when the myocardial cell membrane is damaged during ischemia, hypoxia, etc. The level of cTn in the plasma is elevated in 5–8 h after MI, and the high level could be sustained for 7–10 days, while CKMB is elevated in 4–8 h after MI, and the high level is sustained for 2–3 days (Feng et al., 2008; Yue et al., 2018). Nevertheless, the property of cTn and CKMB for early diagnosis of AMI is weak because the level of these biomarkers elevates late after MI. Thus, a biomarker that could be detected at the early stage of AMI with a better diagnostic value is needed to compensate for the deficiency of cTn and CKMB.

Recently, increasing studies demonstrated that miRNAs were a new era for the management of various diseases, and the role of miRNAs in the timely diagnosis of AMI was promising due to their stability and tissue/cell specificity in peripheral circulation (Mitchell et al., 2008; D'Alessandra et al., 2010; Rupaimoole and Slack, 2017; Li H. et al., 2019; Li P. et al., 2019). Therefore, a systematic review comparing the peak time point and AUC of miRNAs with conventional biomarkers in AMI was performed to clarify the potential diagnostic value of miRNAs in AMI. Among the included studies, most explored the peak time point or AUC of miRNAs, while only a few reported the peak time point and AUC of miRNAs with conventional biomarkers simultaneously.

This study presented that miRNAs might be superior for detection in the early phase of AMI. The consensus that passive leakage from ruptured cells and active secretion through extracellular vesicles derived from stimulated cells are two sources of circulating miRNAs was achieved (Zhang et al., 2013). The miRNAs can be packed in extracellular vesicles and released into the bloodstream when the myocardial cells undergo a microenvironment of ischemia–hypoxia, while cTn leaks into the blood when the myocardial membrane is damaged. Moreover, some miRNA compounds found in cells are more soluble and released into the bloodstream more easily than cTn (Akat et al., 2014). Therefore, miRNAs may be detected earlier than classical biomarkers. In this review, the miR-1-3p, miR-19b-3p, miR-22-5p, miR-122-5p, miR-124-3p, miR-133a, miR-133b, miR-134-5p, miR-150-5p, miR-186-5p, miR-208a, miR-223-3p, miR-483-5p, and miR-499a-5p reached a peak expression earlier than did cTnI/CKMB (D'Alessandra et al., 2010; Białek et al., 2015; Zhang et al., 2015; Cortez-Dias et al., 2016; Wang et al., 2016; Guo et al., 2017; Li H. et al., 2019; Li L. et al., 2019). Furthermore, miR-499a-5p presented as early as 1 h while cTnI and CK-MB were detected 2 h after chest pain in the study by Zhang et al. (2015) These studies indicate that the miRNAs have a short window and might compensate for the deficiency of cTnI/CKMB in the early phase of AMI. However, notably, the peak time of the same miRNAs was diverse in different studies. For example, miR-1-3p and miR-133a reached their peak earlier than cTnI in the study of D'Alessandra et al. (2010) while other studies suggested that the peak time points of miR-1-3p and miR-133a were the same as that of cTnI and CKMB. It is undeniable that the different collection times of the initial blood sample and the different detection time intervals in the included studies might be crucial factors for the peak time pints of miRNAs and the classical biomarkers. The onset time of chest pain symptom provided by patients might not be accurate, which is also a factor that could not be neglected.

Interestingly, this review also showed that most miRNAs possessed satisfactory AUC (0.75–0.99). Among the miRNAs mentioned, miR-1-3p, miR-133a/b, miR-208a/b, and miR-499a-5p got more attention. miR-1, miR-133a, miR-208a/b, and miR-499 are abundantly expressed in the myocardium and involved in various effects associated with heart wounding, arrhythmia, myocardial apoptosis, fibrosis, hypertrophy, and tissue remodeling. In cardiac pathology, including AMI, the expression of miR-1, miR-133a, miR-208a/b, and miR-499 is significantly elevated, and the level of increased cardiac miRNAs in circulation endows miRNAs with the ability of diagnosis for the early phase of AMI (Chistiakov et al., 2016). In this review, most studies reported that the AUC of miR-1, miR-133a/b, miR-208a/b, and miR-499a-5p showed a similar clinical value with that of the traditional biomarkers (Table 2) and that miR-1-3p, miR-133a/b, miR-208a, and miR-499a-5p also presented a short time window (Table 1). The AUC of miRNAs, such as miR-208b and miR-499a-5p, might be more accurate with prolonged detection time, and the diagnostic value is similar to that of hs-cTnT. Moreover, non-AMI diseases, including myopericarditis, acute/chronic heart failure, acute pulmonary embolism, chronic kidney disease, connective tissue disease, and skeletal muscle injury, could lead to a false increase in cTn and CKMB (Feng et al., 2008; Giannitsis and Katus, 2013; Thygesen et al., 2018). Some researches indicated that miR-1-3p, miR-133a, miR-208a/b, and miR-499a-5p might have better specificity than cTn and CKMB, and the specificity could even reach 100%. According to Wang et al. (2010) miR-499a-5p was mainly presented in the heart, and the expression was higher than that in skeletal muscle. Remarkably, miR-208a was only detected in the heart but not in the skeletal muscle. To further clarify the cardiac specificity of miR-208a, Wang et al. (2010) determined the levels of miR-208a in plasma from patients with AMI, acute kidney injury, chronic renal failure, stroke, and trauma. The results demonstrated that miR-208a could only be determined significantly with the highest sensitivity and specificity in AMI patients, but not acute kidney injury, chronic renal failure, stroke, and trauma. The expression level of serum miRNA-499a-5p in patients with stroke, acute and chronic kidney failure, or trauma was also significantly lower than that in patients with AMI (Zhao et al., 2015). Therefore, miRNAs might have optimal specificity to identify AMI from other non-AMI diseases. In addition, this review also showed that combined miRNAs or miRNAs combined with classical biomarkers could provide optimal sensitivity and specificity while enhancing the AUC of single miRNA or classical biomarker. Su et al. (2019) revealed that the combination of miR-1-3p with cTnI improved the sensitivity and the specificity to 86.2 and 90.5%, respectively, which were higher than the sensitivity of cTnI (79.3%) and the specificity of miR-1 (80.2%). These indicated that a combination of miRNAs with traditional biomarkers could compensate for the deficiency of single miRNA and conventional biomarker in sensitivity or specificity. In addition, miR-19a-3p, miR-21-5p, miR-23b-3p, miR-32-5p, miR-92a-3p, miR-134, miR-181a-5p, miR-221-3p, miR-328, miR-494, and miR-1303 also showed a dysregulated level in AMI (Table 2), but their diagnostic value still needs to be verified by a large simple size.

This review further showed that the diagnostic value of miRNAs was associated with the onset time of chest pain, and the miRNAs might show a superior clinical value to traditional biomarkers in patients with AMI within 4 h of the onset of symptoms. In the study by Li L. et al. (2019) the AUC of miR-19b-3p (0.74), miR-223-3p (0.65), and miR-483-5p (0.7) was lower than that of cTnI (0.77) and CKMB (0.73) for diagnosis of AMI (including subjects with chest pain for <3, 3–6, and ≥6 h). Nevertheless, the diagnostic value of these three miRNAs was elevated in subjects with chest pain for <3 h. The AUC for miR-19b-3p, miR-223-3p, and miR-483-5p, and the miRNA panel (a combination of these three miRNAs) was 0.81, 0.76, 0.78, and 0.89, respectively, and each miRNA had better diagnostic accuracy than cTnI (0.68). Meanwhile, the sensitivity of the three miRNAs and the miRNA panel was higher than that of cTnI, and the specificity was also optimal. Furthermore, the positive detection rate of AMI of patients with chest pain for <3 h by miR-19b-3p (76.1%) and the miRNA panel (77.5%) was higher than that of cTnI (47.8%). In the study by Devaux et al. (2012), miR-499a-5p was positive in 93% of patients who presented <3 h after onset of pain while positive expression of hs-cTnT was in 88% of patients. Additionally, miR-1-3p also showed a satisfying diagnostic value compared to the classical biomarkers in subjects within 3 h of the onset of symptoms in the study by Su T. et al. (2020) and Su et al. (2019). Wang et al. (2010) also indicated that miR-208a might have a higher sensitivity than the classical biomarkers in the early AMI stage. All the patients with AMI within 4 h of the onset of symptoms could be detected by miR-208a, while 85% of the cases were detected by cTnI. These phenomena indicated that miRNAs might have better sensitivity than conventional biomarkers in patients with AMI within 4 h of the onset of symptoms. However, in the included studies, most did not record the exact onset time of chest pain or classified the patients according to the time of chest pain, which might be a critical factor for the different trend of diagnostic value of the same miRNAs.

Overall, miR-1-3p, miR-19b-3p, miR-133a/b, miR-208a/b, miR-223-3p, miR-483-5p, and miR-499a-5p may be more valuable than classical biomarkers for the early diagnosis of AMI, and these miRNAs show a short time window within 4 h of the onset of symptoms and satisfactory sensitivity and specificity, except miR-133a/b and miR-208b. Combined miRNAs or miRNAs combined with traditional biomarkers could also compensate for the deficiency of single miRNA or traditional biomarker with respect to sensitivity or specificity for an optimal clinal value. Nonetheless, some confounders and limitations of this review should be considered due to the restricted reports.

### Potential Confounders and Limitations

Subjects, sample size, the collecting time of samples, and quantitative methods of miRNAs not only were the main potential confounders and limitations of this systematic review but also influenced the inconsistency of the level, peak time, and statistical significance of the same miRNAs.

### Population-Based Confounders

For the sample size of included studies, 27/40 studies enrolled subjects with a case group of <100. Ten and three studies had case group sample sizes of 100–300 and ≥300, respectively. miR-1-3p, miR-19b-3p, miR-133a/b, miR-208a/b, miR-223-3p, miR-483-5p, and miR-499a-5p were relatively reliable, powerful biomarkers based on multiple normalized researches and had larger sample sizes than other miRNAs. Although the number of subjects of the case group in the studies of miR-19a-3p, miR-23b-3p, miR-181a-5p, etc. was weak, the results of these studies still showed a meaningful clue that these miRNAs might be potential biomarkers but need to be verified by large samples in future studies.

### Sampling Confounders

The determination of miRNAs from different biological fluids remains controversial. Hermenegildo et al. (2017) reported that miRNA expressions varied according to the sample types from NSTEMI patients. Mompeón et al. (2020) indicated that samples from serum or plasma could be contaminated by red cells, white blood cells, platelets, and hemolysis and proposed that serum was preferable in circulating miRNA studies than plasma and that platelet-poor plasma would be rather appropriate when the miRNAs were highly expressed in the platelets. However, most of the studies selected plasma for miRNA determination but did not show whether the sample type influenced the levels of the selected miRNAs. Although the trends of the same miRNA results did not differ markedly between plasma and serum, it was still a potential factor for the inconsistency of the levels, peak time, and statistical significance of the same miRNAs. Moreover, the different initial sample collection time points and detection intervals were also major confounders that should not be neglected.

### Detection Method Confounders

The majority of the included studies used qPCR to detect miRNAs while one study used droplet digital PCR (ddPCR) to detect miRNAs. The methods of qPCR are complex and time-consuming. Thus, a novel technology to reduce processing steps and improve the efficiency of detection needs to be addressed in the future. Besides that, ddPCR can absolutely quantitate nucleic acids with greater reproducibility and less inter- and intra-assay variability compared to qPCR (Hindson et al., 2011, 2013; Robinson et al., 2018). Robinson et al. (2018) indicated that the calculation method of the PCR value might influence the diagnostic accuracy of miRNAs and that ddPCR was superior in both technical proficiency and diagnostic potential compared to qPCR. Also, ddPCR was preferred for accurate and reproducible quantification of miRNAs in cardiovascular biology, which provided a reference for future research.

## Conclusions

miR-1-3p, miR-19b-3p, miR-133a/b, miR-208a/b, miR-223-3p, miR-483-5p, and miR-499a-5p are shown to be more valuable than classical biomarkers for early diagnosis of AMI; particularly, miR-1-3p, miR-19b-3p, miR-208a, miR-223-3p, miR-483-5p, and miR-499a-5p appear to have the most potential as biomarkers in patients with AMI within 4 h of the onset of symptoms due to their short time window and optimal sensitivity and specificity. However, the diagnostic value of miRNAs and classical biomarkers in patients with AMI at different periods from the onset of chest pain needs further substantiation using large samples. A novel technology to improve the detection efficiency of miRNAs is also needed.

## Data Availability Statement

The original contributions presented in the study are included in the article/Supplementary Material, further inquiries can be directed to the corresponding author/s.

## Author Contributions

BW, YL, and XHao: theme and design of the research. JY, YT, and LM: verification of data. XHan, DW, HL, JY, and TL: statistical analysis. BW and YL: writing of the manuscript. MZ and XW: critical revision of the manuscript for intellectual content and obtaining funding. All authors contributed to the article and approved the submitted version.

## Conflict of Interest

The authors declare that the research was conducted in the absence of any commercial or financial relationships that could be construed as a potential conflict of interest.

## Abbreviations

AMI: acute myocardial infarction
CHD: coronary heart disease
cTn: cardiac troponin
CKMB: creatine kinase MB
hs-cTnT: high-sensitivity cardiac troponin T
AUC: area under the curve
NSTEMI: non-ST segment elevation myocardial infarction
STEMI: ST-segment elevation myocardial infarction.
