# Supplementary material for: Comparison of the Clinical Value of miRNAs and Conventional Biomarkers in AMI: A Systematic Review
Source: Front Genet. 2021 Jun 17;12:668324. doi: 10.3389/fgene.2021.668324 (PMC8248539; doi:10.3389/fgene.2021.668324)
Supplement: Supplementary file 1 [file Data_Sheet_1.docx]

**Table S1. Standardization of miRNAs names**

| **miRNAs names in the included studies** | **miRNAs names after standardization** |
| --- | --- |
| let-7b (17) | let-7b-5p* |
| miR-1 (6,16,21,28,30,31,32,33,34,43) | miR-1-3p* |
| miR-122-5p (15,49) | miR-122-5p* |
| miR-124 (12) | miR-124-3p* |
| miR-126 (16) | miR-126-3p* |
| miR-126-3p (26) | miR-126-3p* |
| miR-1303 (42) | miR-1303* |
| miR-132-5p (13) | miR-132-5p* |
| miR-133 (21) | miR-133# |
| miR-133a-3p (15) | miR-133a-3p* |
| miR-133a (6,19,31,33,38,39) | miR-133a# |
| miR-133b (6,15) | miR-133b* |
| miR-134 (32,40) | miR-134# |
| miR-134-5p (14) | miR-134-5p* |
| miR-1-3p (15) | miR-1-3p* |
| miR-150-5p (13) | miR-150-5p* |
| miR-181a (27) | miR-181a-5p* |
| miR-186 (32) | miR-186-5p* |
| miR-186-5p (14) | miR-186-5p* |
| miR-195 (17) | miR-195-5p* |
| miR-19a (24) | miR-19a-3p* |
| miR‐19b (46) | miR‐19b-3p* |
| miR-19b-3p (14) | miR‐19b-3p* |
| miR-208 (21,32,42) | miR-208a-3p* |
| miR-208a (11,31,35) | miR-208a# |
| miR-208b (20,23,33,43,44) | miR-208b# |
| miR-208b-3p (15) | miR-208b-3p* |
| miR-21 (29,35) | miR-21-5p* |
| miR-21-5p (48) | miR-21-5p* |
| miR-221-3p (41) | miR-221-3p* |
| miR-223 (32,46) | miR-223-3p* |
| miR-22-5p (13) | miR-22-5p* |
| miR-23b (25) | miR-23b-3p* |
| miR-30a (17) | miR-30a-5p* |
| miR-32-5p (36) | miR-32-5p* |
| miR-328 (40) | miR-328# |
| miR-361-5p (48) | miR-361-5p* |
| miR‐483‐5p (46) | miR‐483‐5p* |
| miR-494 (42) | miR-494# |
| miR-497 (18) | miR-497-5p* |
| miR-499 (20,21,22,23,31,32,33,35,42,43,44,45,47) | miR-499a-5p* |
| miR-499-5p (6,15) | miR-499a-5p* |
| miR-519e-5p (48) | miR-519e-5p* |
| miR-92a (37) | miR-92a-3p* |

Note: The names of all miRNAs in the included studies were standardized through the miRBase database. *: The names of standardized miRNAs base on the miRBase database; #:The names of the miRNAs were the original names in the included studies because the miRNAs primers were not clear in the original study and the standardized names could not be matched in the miRBase database.

**Table S2. Patient Characteristics**

| **Study ID** | **Medical history of control group** | | | | **Medical history of case group** | | | | **Age(y）** | | **Sample** | |
| --- | --- | --- | --- | --- | --- | --- | --- | --- | --- | --- | --- | --- |
|  | **hypertension** | **diabetes** | **hyperlipidemia** | **smoking** | **hypertension** | **diabetes** | **hyperlipidemia** | **smoking** | **control group** | **case group** | **miRNAs** | **cTn/CKMB** |
| D'Alessandra, Y(6) | 3(17.6%) | 1(5.9%) | 2(11.8%) | 8(47%) | 3(37.5%) | 2(25%) | 4(50%) | 3(37.5%) | 46.1±13.9 | 61.9±9 | plasma | plasma |
| Białek, S(11) | 7(87.5%) | 1(12.5%) | 6(75%) | / | 7(36.8%) | 3(15.8%) | 13(68.4%) | / | 60(58-67) | 58(55-65) | plasma |  |
| Guo, M. L(12) | / | / | / | 9(20%) | / | / | / | 15(16.7%) | 36.2±5.2 | 35.2±5.6 | serum | serum |
| Li, H(13) | 12(22%) | 6(11%) | 14(25%) | 15(27%) | 11(31%) | 5(14%) | 23(37%) | 18(51%) | 56.36±12.36 | 60.86±11.25 | plasma | plasma |
| Wang, K.J(14) | 5(25%) | 1(5%) | / | 7(35%) | 9(50%) | 3(17%) | / | 9(50%) | 54.36±11.3 | 57.86±10.75 | plasma | plasma |
| Cortez-Dias, N(15) | / | / | / | / | / | / | / | / | / | / | serum | serum |
| Long G/2012b(16) | 8(30%) | 1(4%) | 2(8%) | 11(55%) | 8(50%) | 2(12%) | 2(12%) | 12(75%) | 51±12.3 | 53±12.5 | plasma | plasma |
| Long G/2012a(17) | 9(30%) | 2(6%) | 1（3%） | 20(67%) | 9（50%） | 3(16%) | 2（11%） | 14（78%) | 50±12.3 | 55±11.4 | plasma | plasma |
| Li Z(18) | 9 (29%) | 3 (10%) | / | / | 12 (44%) | 4 (15%) | / | / | 51.21±12.25 | 54.15±11.34 | plasma | plasma |
| Wang F(19) | / | / | / | / | / | / | / | / | 50.37±11.45 | 54.15±12.54 | plasma | plasma |

**Table S3. Patient Characteristics**

| **Study ID** | **Medical history of control group** | | | | **Medical history of case group** | | | | **Age(y)** | | **Sample** | |
| --- | --- | --- | --- | --- | --- | --- | --- | --- | --- | --- | --- | --- |
|  | **hypertension** | **diabetes** | **hyperlipidemia** | **smoking** | **hypertension** | **diabetes** | **hyperlipidemia** | **smoking** | **control group** | **case group** | **miRNAs** | **cTn/CKMB** |
| Agiannitopoulos, K(20) | 14(28%) | / | 17（34%） | 35(70%) | 23(28.75%) | / | 20（25%） | 32(64%) | 59.30±9.82 | 62.12±10.99 | plasma | / |
| Liu, G(21) | 0(0.00%) | 0(0.00%) | 0(0.00%) | 14(46.67%) | 97(66.90%) | 63(43.45%) | 107(73.80%) | 79(54.48%) | 65 | 67 | plasma | plasma |
| Vengatapathy, K. V(22) | / | / | / | / | / | / | / | / | 30-60 | 30-60 | serum | serum |
| Devaux, Y/2012(23) | 17(20.00%) | 0(0.0%) | 12(14.00%) | 20(23.00%) | 235(46.00%) | 116(23%) | 217(43.00%) | 207(41%） | 53(40-60) | 62(20-91) | plasma | / |
| Zhong, J(24) | / | / | / | / | / | / | / | / | 58.64 | 60.13 | plasma | plasma |
| Zhang, J(25) | 32(53.3%) | 19(31.7%) | 31(51.7%) | 24(40%) | 50(62.5%) | 31(38.8%) | 48(60%) | 39(48.8%) | 63.15±7.52 | 62.83±7.52 | plasma | serum |
| He, Y(26) | / | / | / | / | / | / | / | / | / | / | plasma | plasma |
| Zhu, J(27) | 27(45%) | 12(20%) | 28(46.6%) | 39(65%) | 26(43.3%) | 15(25%) | 29(48.3%) | 38(63.3%) | 63.2±12.2 | 64.1±10.3 | plasma | serum |
| Su, T/2019a(28) | / | / | / | / | / | / | / | / | / | / | plasma | serum |
| Zhang, Y/2016(29) | 4(40%) | 1(10%) |  | 3(30%) | 11(68.75%) | 4(23.53%) |  | 12(70.59%) |  |  | plasma | plasma |
| Su, T/2019b(30) | 119(71.26%) | 45(29.45%) |  | 47(28.14%) | 127(72.99%) | 53(30.46%) |  | 116(66.67%) | 67(60,75) | 68(55,75) | plasma | serum |
| Wang, G. K(31) | 14(42.4%) | 7(21.2%) | 12(36.4%) | 13(39.4%) | 17(51.5%) | 8(24.2%) | 20(60.6%) | 12(36.4%) | / | 63.5±10.1 | plasma | serum |
| Li, C(32) | 53(53%) | 12(12%) | / | / | 69(58.97%) | 29(24.79%) | / | / | 61.10±7.79 | 62.70±11.40 | serum | serum |
| Li, Y. Q(33) | 15(46.88%) | 5(15.63%) | 14(43.75%) | 13(40.63%) | 38(56.72%) | 13(19.4%) | 36(53.75%) | 32(47.76%) | 61.75±9.58 | 63.84±11.17 | plasma | plasma |
| Li, L. M(34) | 13(46.43%) | 3(10.71%) | 12(42.86%) | 12(48.86%) | 33(58.93%) | 10(17.86%) | 30(53.57%) | 27(48.21%) | 60.5±9.1 | 63.95±11.34 | plasma | plasma |
| Robinson, S(35) | / | / | / | / | / | / | / | / | / | / | serum | / |
| Dai, Y(36) | 28(56.00%) | 14(28.00%) | 25(50.00%) | 26(52.00%) | 47(53.41%) | 27(30.68%) | 46(52.27%) | 46(52.27%) | 60.82±18.13 | 60.81±17.35 | serum | / |
| Zhang, Y/2017(37) |  | 20(5.7%) |  | 7(20%) |  | 8(19%) |  | 10(23.8%) | 52.6±5.1 | 52.8±5.6 | plasma | plasma |
| Jia, K. G(38) | 56(70.89%) | 23(29.11%) | / | 134(57.51%) | 142(60.94%) | 65(27.9%) | / | 29(36.71%) | / | / | plasma | plasma |
| Yuan, L(39) | 67(60.91%) | 51(46.36%) | 32(29.10%) | / | 65(63.73%) | 64(62.75%) | 39(38.24%) | / | 66±14 | 65±12 | plasma | plasma |
| He,F(40) | 9(30%) | 3(10%) | 6(20%) | 13(43%) | 172(48%) | 57(16%) | 126(35%) | 190(53%) | 57±10 | 58±14 | plasma | plasma |
| Coskunpinar, E(41) | 10(62.5%) | 7(43.75%) | 10(62.5%) | 9(56.25%) | 19(70.37%) | 9(33.33%) | 19(70.37%) | 13(48.15%) | 51.8 ± 9.3 | 57.2 ± 9.7 | plasma | / |
| Li, P(42) | 1(10%) | 0(0.00%) | 5(50%) | 1(10%) | 24(58.53%) | 9(21.95%) | 26(53.41%) | 16(39.02%) | 59.5±12.87 | 62.95±11.04 | plasma | / |
| Gidlof, O(43) | / | / | / | / | / | / | / | / | / | / | plasma | plasma |
| Devaux, Y/2015(44) | 583(63%) | 151(16%) | / | 547(59%) | 179(80%) | 57(26%) | / | 136(60%) | 61(49-74) | 72(61-80) | plasma | / |
| Zhao, C. H(45) | / | / | / | / | / | / | / | / | 61.9±12.1 | 60.1 ± 11.3 | serum | serum |

**Table S4. Patient Characteristics**

| **Study ID** | **Medical history of control group** | | | | **Medical history of case group** | | | | **Age(y）** | | **Sample** | |
| --- | --- | --- | --- | --- | --- | --- | --- | --- | --- | --- | --- | --- |
|  | **hypertension** | **diabetes** | **hyperlipidemia** | **smoking** | **hypertension** | **diabetes** | **hyperlipidemia** | **smoking** | **control group** | **case group** | **miRNAs** | **cTn/CKMB** |
| Li, L(46) | 62(44.3%) | 33(23.6%) | 18(12.9%) | 14(10%) | 82(58.6%) | 34(24.3%) | 50(35.7%) | 71(50.7%) | 62.83±16.31 | 63.34±13.77 | plasma | plasma |
| Zhang, L(47) | 59(69.4%) | 29(34.1%) | 18(21.2%) | 31(36.5%) | 91(64.08%) | 40(28.17%) | 37(26.06%) | 79(55.6%) | 66.54±10.61 | 64.86±12.84 | plasma | serum |
| Wang, F(48) | 9 (52.9%) | 1 (5.9%) | / | 9 (52.9%) | 4 (14.4%) | 0 (0.0%) | / | 5 (17.8%) | 58±11 | 52±11 | plasma | plasma |
| Yao, X. L(49) | / | 18(46.15%) | / | 23(58.97%) | / | 21(42.00%) | / | 29(0.58%) | 62.7±10.5 | 63.2±11.4 | plasma | plasma |

Note：In the study of Li, L[46], 10 patients with AMI were researched for peak level of miRNAs and classical biomarkers. 140 non-AMI and 140 patients with AMI were researched for AUC comparison of miRNAs and classical biomarkers (subjects with chest pain＜3h, 3h-6h, ≥6h). 48 non-AMI and 71 patients with AMI were researched for AUC comparison of miRNAs and classical biomarkers (subjects with chest pain less than 3h). All of the subjects included in case size were displayed in Table S4.

In the study of Zhang, L[47], 142 patients with AMI were researched for peak level of miRNAs and classical biomarkers. 85 non-AMI and 142 patients with AMI were researched for AUC comparison of miRNAs and classical biomarkers. All of the subjects included in case size were displayed in Table S4.

In the study of Wang, F[48], 6 patients with AMI were researched for peak level of miRNAs and classical biomarkers. 12 healthy volunteers and 9 patients with AMI were researched for AUC comparison of miRNAs and classical biomarkers. All of the subjects included in case size were displayed in Table S4.

In the study of Yao, X. L[49], 50 patients with AMI were researched for peak level of miRNAs and classical biomarkers. 39 non-AMI and 50 patients with AMI were researched for AUC comparison of miRNAs and classical biomarkers. All of the subjects included in case size were displayed in Table S4.

**Table S5.** **Newcastle–Ottawa Scale (NOS) of included studies**

| **No** | **Author** | **Year** | **Method** | **Sample Size** | **Score** |
| --- | --- | --- | --- | --- | --- |
| 1 | D'Alessandra, Y(6) | 2010 | observational research | 25 | 8 |
| 2 | Białek, S(11) | 2015 | observational research | 27 | 6 |
| 3 | Guo, M. L(12) | 2017 | observational research | 135 | 7 |
| 4 | Li, H(13) | 2019 | observational research | 90 | 7 |
| 5 | Wang, K.J(14) | 2016 | observational research | 38 | 8 |
| 6 | Cortez-Dias, N(15) | 2016 | observational research | 58 | 6 |
| 7 | Long G(16) | 2012b | observational research | 42 | 7 |
| 8 | Long G(17) | 2012a | observational research | 48 | 7 |
| 9 | Li Z(18) | 2014 | observational research | 58 | 7 |
| 10 | Wang F(19) | 2013 | observational research | 40 | 8 |
| 11 | Agiannitopoulos, K(20) | 2018 | observational research | 130 | 8 |
| 12 | Liu, G(21) | 2018 | observational research | 175 | 8 |
| 13 | Vengatapathy, K.V(22) | 2019 | observational research | 120 | 7 |
| 14 | Devaux, Y (23) | 2012 | observational research | 597 | 6 |
| 15 | Zhong, J(24) | 2014 | observational research | 301 | 6 |
| 16 | Zhang, J(25) | 2018 | observational research | 140 | 6 |
| 17 | He, Y(26) | 2017 | observational research | 57 | 7 |
| 18 | Zhu, J(27) | 2016 | observational research | 120 | 8 |
| 19 | Su, T(28) | 2019a | observational research | 337 | 7 |
| 20 | Zhang, Y(29) | 2016 | observational research | 27 | 8 |
| 21 | Su, T(30) | 2019b | observational research | 341 | 7 |
| 22 | Wang, G. K(31) | 2010 | observational research | 66 | 6 |
| 23 | Li, C(32) | 2013 | observational research | 217 | 8 |
| 24 | Li, Y. Q(33) | 2013 | observational research | 99 | 7 |
| 25 | Li, L. M(34) | 2014 | observational research | 84 | 8 |
| 26 | Robinson, S(35) | 2018 | observational research | 44 | 6 |
| 27 | Dai, Y(36) | 2020 | observational research | 138 | 8 |
| 28 | Zhang, Y(37) | 2017 | observational research | 77 | 7 |
| 29 | Jia, K. G(38) | 2016 | observational research | 312 | 7 |
| 30 | Yuan, L(39) | 2016 | observational research | 212 | 7 |
| 31 | He,F(40) | 2014 | observational research | 389 | 8 |
| 32 | Coskunpinar, E(41) | 2016 | observational research | 43 | 7 |
| 33 | Li, P(42) | 2019 | observational research | 51 | 7 |
| 34 | Gidlof, O(43) | 2013 | observational research | 407 | 6 |
| 35 | Devaux, Y(44) | 2015 | observational research | 1155 | 6 |
| 36 | Zhao, C. H(45) | 2015 | observational research | 119 | 8 |
| 37 | Li, L(46) | 2019 | observational research | 280 | 7 |
| 38 | Zhang, L(47) | 2015 | observational research | 227 | 7 |
| 39 | Wang, F(48) | 2014 | observational research | 45 | 6 |
| 40 | Yao, X. L(49) | 2015 | observational research | 89 | 7 |
